# Thermal and mechanical characterization of nanoporous two-dimensional MoS_2_ membranes

**DOI:** 10.1038/s41598-022-11883-5

**Published:** 2022-05-11

**Authors:** Van-Trung Pham, Te-Hua Fang

**Affiliations:** 1 Department of Mechanical Engineering, National Kaohsiung University of Science and Technology, Kaohsiung, 807 Taiwan; 2 Department of Mechanical Engineering, Pham Van Dong University, Quang Ngai, 570000 Vietnam

**Keywords:** Theory and computation, Materials science, Nanoscale materials, Two-dimensional materials

## Abstract

For practical application, determining the thermal and mechanical characterization of nanoporous two-dimensional MoS_2_ membranes is critical. To understand the influences of the temperature and porosity on the mechanical properties of single-layer MoS_2_ membrane, uniaxial and biaxial tensions were conducted using molecular dynamics simulations. It was found that Young’s modulus, ultimate strength, and fracture strain reduce with the temperature increases. At the same time, porosity effects were found to cause a decrease in the ultimate strength, fracture strain, and Young’s modulus of MoS_2_ membranes. Because the pore exists, the most considerable stresses will be concentrated around the pore site throughout uniaxial and biaxial tensile tests, increasing the possibility of fracture compared to tensing the pristine membrane. Moreover, this article investigates the impacts of temperature, porosity, and length size on the thermal conductivity of MoS_2_ membrane using the non-equilibrium molecular dynamics (NEMD) method. The results show that the thermal conductivity of the MoS_2_ membrane is strongly dependent on the temperature, porosity, and length size. Specifically, the thermal conductivity decreases as the temperature increases, and the thermal conductivity reduces as the porosity density increases. Interestingly, the thermal and mechanical properties of the pristine MoS_2_ membrane are similar in armchair and zigzag directions.

## Introduction

Since the discovery of graphene in 2004^1^, two-dimensional (2D) materials have become one of the research focuses in the fields of physics, chemistry, and material science^2–6^. Recently, molybdenum disulfide (MoS_2_), as a prototypical example of transition metal dichalcogenide (TMDs), has attracted considerable interest from scientists due to its unique properties. It is constituted of an S-Mo-S sandwich structure, with each layer of molybdenum (Mo) and sulfur (S) atoms forming a regular hexagon. Between the layers, only weak van der Waals forces connect them^7^. In addition to its traditional use as a solid lubricant, it is well known as a new direct-gap semiconductor^8^. As a result of its unique mechanical and electronic characteristics, MoS_2_ is considered a promising material in a series of applications such as field-effect transistors, flexible electronic components, photoelectric devices, nanomechanical resonators, and lubricating materials^9–13^. The structure and properties of MoS_2_ have been studied to assist in designing and fabricating these devices. Zhao et al.^14^ reported that for 2D MoS_2,_ the most stable are 1H-MoS_2_ and 1T′-MoS_2_ phases. Moreover, DFT calculations show that 1H-MoS_2_ is more stable than 1T′-MoS_2_^15,16^. Due to the 1H-MoS_2_ phase being the most stable configuration and the most popular in nature, so this study focuses on this structure.

In order to the synthesis of monolayer MoS_2_, both bottom-up and top-down techniques are widely applied^17–20^. Moreover, many studies point out that pore engineering can provide a viable tool to tune the material behavior of stretchable and flexible devices that require specific thermomechanical characteristics from precise control of point defects^21–23^. Nanoporous two-dimensional materials have been used in various applications, including energy generation and storage^24^. The quantitative studies of the effect of these defects on thermal and mechanical characteristics in monolayer MoS_2_ are critical. It can provide effective guidance on intentionally manufactured to adjust the characteristics of MoS_2_ to suit the desired functionalities in the application. Recently, studies on the influence of defects on mechanical properties as well as thermal conductivity have received much attention. For instance, the buckling behaviour of rectangular MoS_2_ nanoribbons with defects subjected to axial compression was analyzed by Yao et al.^25^. The propagation of nano cracks in single-layer MoS_2_ was investigated by Bao et al.^26^. Islam et al.^27^ discovered the effect of vacancy defect and doping on mechanical properties; moreover, the effect of grain boundary was also investigated. Other authors have also studied the impact of the intrinsic structural defect and engineered defects on the mechanical characteristic of monolayer MoS_2_^28,29^. Besides the studies on the mechanical properties of the MoS_2_ sheet, there are many studies on its thermal conductivity. For instance, many experimental works^30–34^ and numerical simulations^35–40^ have been conducted to measure the thermal conductivity of monolayer MoS_2_. The measured result is 40.8 W/m K based on the optothermal Raman technique^31^. Yan et al.^32^ reported the thermal conductivity value obtained from Raman spectra is 34.5 W/m K. Jiang et al.^33^ and Zhang et al.^34^ measured the thermal conductivity to be 82.0 W/m K and 84 W/m K for the single-layer MoS_2_. In numerical simulations, Xiang et al.^35^ and Gu et al.^36^ used first-principles to compute the thermal conductivity of monolayer MoS_2_ was around 47.50 W/m K, and 138 W/m K, respectively. Based on NEMD and EMD simulations, Liu et al.^37^ have been reported that phonon thermal conductivities of monolayer MoS_2_ are 1.70 W/m K and 1.35 W/m K at room temperature. Using a similar method, two research groups^38,39^ obtained different thermal conductivity values of 23.2 W/m K and 101.43 W/m K, respectively. Despite multiple investigations of monolayer MoS_2_ thermal conductivity, considerable discrepancies in thermal conductivity values were discovered owing to variances in techniques, force fields, and so on. Therefore, it is necessary to study the thermal conductivity of monolayer MoS_2_ further. Nevertheless, the effect of nanopores uniformly distributed on the single-layer MoS_2_ as well as the effect of temperature has rarely been investigated. The effect of porosity and temperature on the biaxial tensile test, in particular, has not been sufficiently explored. Hence, studying their influence on the mechanical and thermal characterization of nanoporous 2D MoS_2_ membranes is extremely desirable.

Motivated by the above discussion, we employ the molecular dynamics method to investigate the effects of temperature and porosity on the mechanical behaviours of the MoS_2_ membrane through uniaxial and biaxial tensions. Moreover, the impact of temperature and porosity on the thermal conductivity of the MoS_2_ membrane was studied by non-equilibrium MD (NEMD) simulations.

## Results

**Figure 1:**
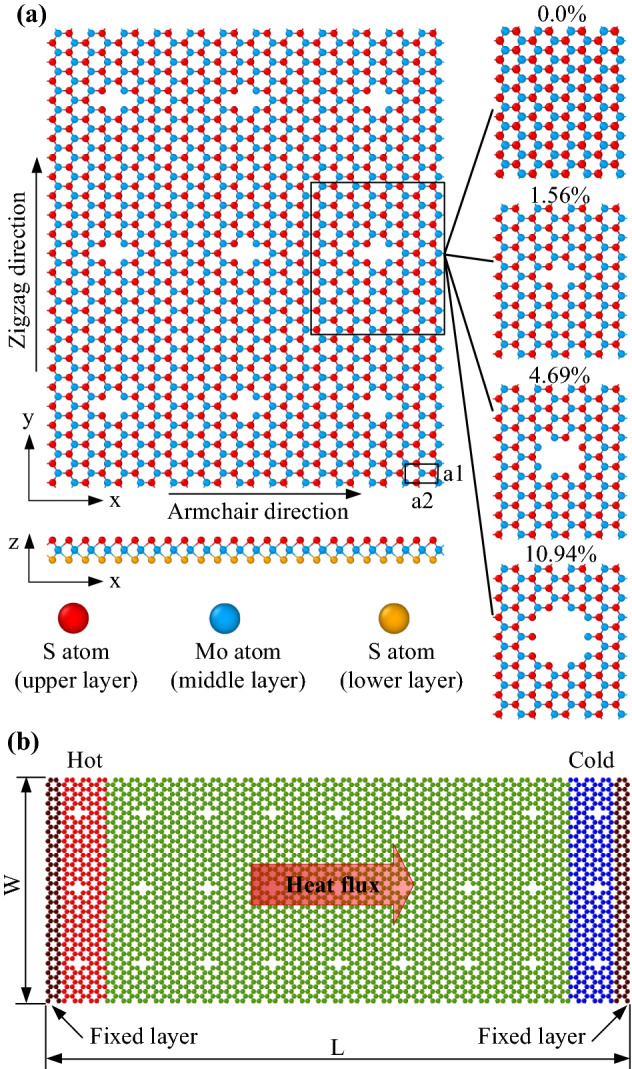
The structures of monolayer MoS_2_ membranes with various porosities (**a**) setup for the uniaxial and biaxial tensions, (**b**) schematic illustration of the NEMD thermal conductivity calculations of monolayer MoS_2_.

A model of porous monolayer MoS_2_ with various porosities was utilized for the biaxial and uniaxial tensions, as presented in Fig. 1a, to explore the impact of temperature and porosity on the mechanical properties of nanoporous two-dimensional MoS_2_ membranes. The nanopores are evenly dispersed over the membrane, where the ratio of the missing atom in the nanopores to the total number of atoms in a perfect MoS_2_ nanosheet is defined as the porosity. In this study, we investigate the mechanical characteristics of MoS_2_ with various porosities of 0% (pristine sheet), 1.56%, 4.69%, and 10.94%. To determine the thermal conductivity of MoS_2_ membranes, non-equilibrium molecular dynamics (NEMD) simulations were performed. The schematic illustration of the NEMD thermal conductivity calculations of the monolayer MoS_2_ membrane is displayed in Fig. 1b. The dimension of the monolayer MoS_2_ sample is *W × L*, with *W* and *L* are the width of the sample and the dimension of the heat transfer direction, respectively. The porosity of the sample is the same as the model in Fig. 1a. The thermal conductivity values obtained from the NEMD method are significantly affected by the length of samples^41,42^. Thus, the width of the chosen sample is *W* = 7.48 nm, while *L* is varied in the range of 19.4, 30.2, 41.0, 60.5, and 101.5 nm in the armchair direction, and 19.96, 29.94, 39.92, 59.88, 99.80 nm in the zigzag direction. The length size along the two directions, zigzag and armchair, has a small deviation due to the lattice constants *a*_*1*_ and *a*_*2*_ in the rectangular unit cell being different.

### Uniaxial tension

**Figure 2:**
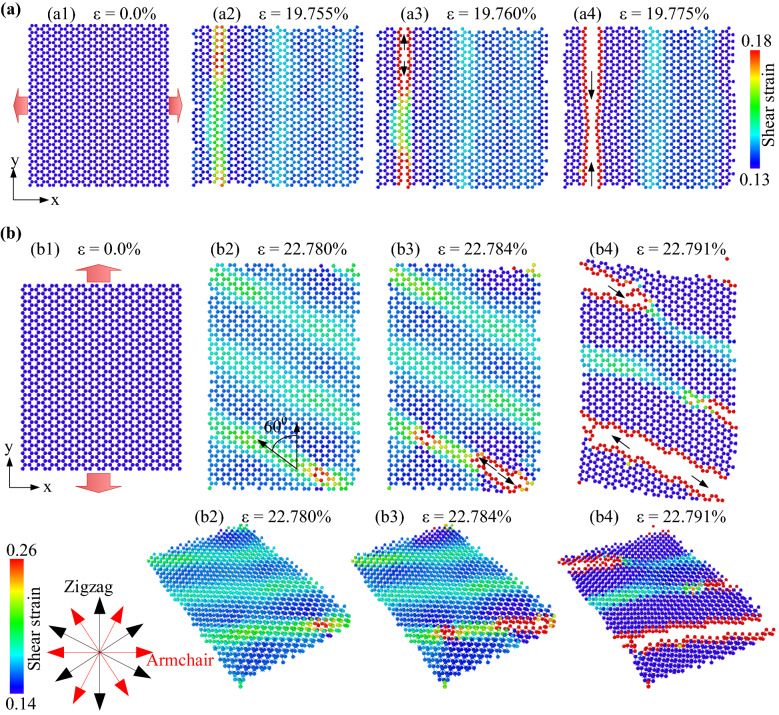
The atomic shear strain evolution and deformation behavior of monolayer MoS_2_ membrane under tension (**a**) along the *x* (armchair) direction at 1 K, (**b**) along the *y* (zigzag) direction at 1 K.

In the uniaxial tensile test at 1 K, the atomic shear strain evolution and deformation behaviour of monolayer MoS_2_ membrane along the x (armchair) direction are shown in Fig. 2a. We observed that as the strain rises, the shear strain in the membrane rises. The membrane begins to crack when the strain gets to 19.755%. The fracture then spreads quickly in a direction perpendicular to the tensile direction; the membrane is entirely broken when the strain reaches 19.775%. The black arrows show the crack propagation, and the result shows the crack propagation along the zigzag edge. Figure 2b shows the von Mises shear strain evolution and deformation behaviour of monolayer MoS_2_ membrane under tension along the *y* (zigzag) direction at 1 K. As seen in Fig. 2b2, a local plastic deformation band at an angle of 60° is produced under zigzag loading. The shear strain distribution on each atom was used to distinguish this plastic deformation band. The results show that when the strain is 22.780%, the MoS_2_ membrane will begin to appear cracks. With a further increment in the tensile strain, these cracks quickly develop until the membrane is totally destroyed, when the strain has a value of 22.791%. Unlike uniaxial tension in the armchair direction, cracks in uniaxial tension in the zigzag direction propagate in the direction of the black arrow, not perpendicular to the direction of tensing. It is interesting to see that the direction of the black arrow also runs along the edge of the zigzag. This shows that under uniaxial tension loading, the crack tends to propagate along the zigzag edge, implying that the zigzag's edge energy is lower than the armchair's edge energy. In a recently published study, Islam et al.^27^ explained the crack mechanism that preferentially propagates along the zigzag edge, which is consistent with the previous experimental study^43^ for MoS_2_ material.

In the uniaxial tensile test at 1 K, the atomic shear strain evolution and deformation behaviour of monolayer MoS_2_ membranes with different porosities along the x (armchair) direction and y (zigzag) direction are shown in Supplementary Figs. 1, 2. We observed that as the strain rises, the shear strain in the membrane rises. Due to the existence of holes, stress is highly concentrated around the positions of the holes in tension, leading as the strain increases, the atomic bonds at these positions will break first. Also, due to the concentration of stress at these positions, the membrane becomes weaker, leading to decreased stress required for their failure. Therefore, the ultimate strength and failure strain of the porosity sheets are decreased compared to the pristine sheet. The cracks begin to appear and spread rapidly in the direction of the pink arrow. The crack propagation direction is perpendicular to the tensile direction.

**Figure 3:**
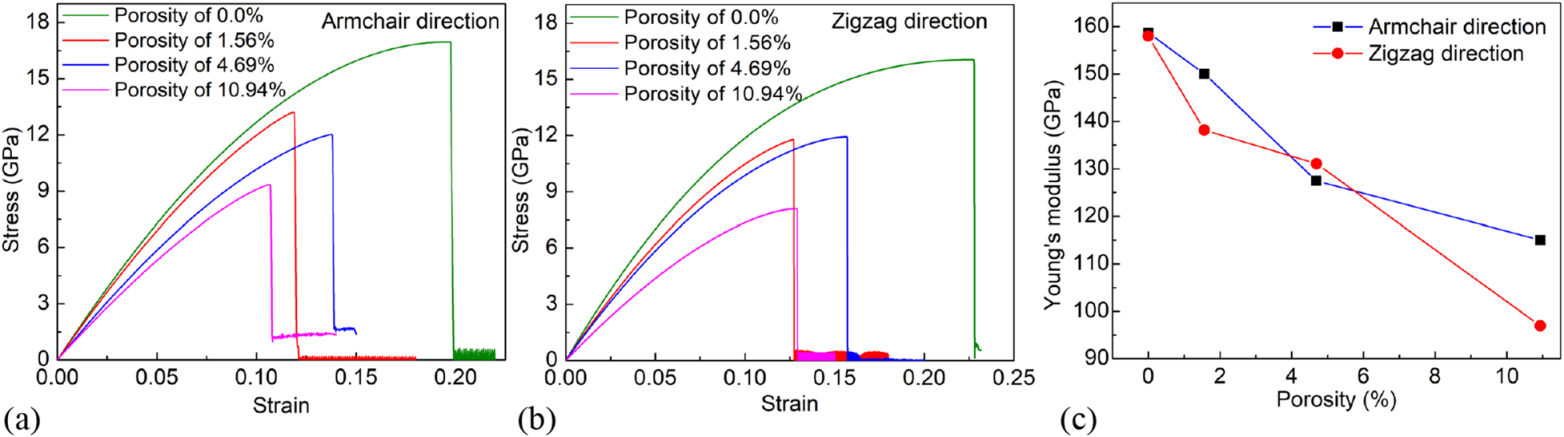
(**a**) Tensile stress–strain curves of monolayer MoS_2_ membrane under tension along the armchair direction with various porosities, where *σ* is the normal stress. (**b**) Tensile stress–strain curves of monolayer MoS_2_ membrane under tension in the zigzag direction with various porosities, where *σ* is the normal stress. (**c**) The porosity dependence of Young’s modulus.

Figure 3 depicts the mechanical properties of the MoS_2_ membrane under uniaxial tensile test at 1 K for various porosities. Figure 3a shows the tensile stress–strain (*σ-ε*) curves of monolayer MoS_2_ membranes under uniaxial tensile test in the direction of the armchair with various porosities, where *σ* is the normal stress (*σ*_*xx*_). The result points out that the ultimate strength values of MoS_2_ membranes with porosities of 0% (pristine sheet), 1.56%, 4.69%, and 10.94% are 16.96, 13.21, 12.03, and 9.32 GPa, respectively. Figure 3b shows the stress–strain curves of the MoS_2_ membrane under the uniaxial tensile test in the direction of the zigzag, where *σ* is the normal stress (*σ*_*yy*_). The result indicates that the ultimate strength values of MoS_2_ sheets with porosities of 0% (pristine sheet), 1.56%, 4.69%, and 10.94% are 16.25, 11.83, 11.80, and 8.10 GPa, respectively. The result points out that the total hole length for the sheets with a porosity of 1.56%, and 4.69% in the armchair direction is equal, leading to ultimate strength values being approximately in two membranes under uniaxial tension in armchair direction. From the tensile stress–strain curves, Young’s modulus values of the membranes are calculated and depicted in Fig. 3c. The results indicate that as porosity raises, ultimate strength decreases. However, the fracture strain value does not seem to be correspondingly sensitive to the porosity of the material, which is similar to previous studies for graphene sheets, borophene^22,44^. The result demonstrates that Young’s modulus, ultimate strength, and failure strain of the pristine MoS_2_ membrane when tensile loading in the zigzag and armchair directions are almost the same. This shows that the pristine MoS_2_ membrane is nearly isotropy in mechanical properties. For the sheets with a porosity of 4.69%, the total hole length in the armchair direction is approximately the total hole length in the zigzag direction. Then, failure strain, ultimate strength, and Young’s modulus values are approximately in two directions. For the membranes with a porosity of 1.56% and 10.56%, the values of ultimate stress, fracture strain, and Young’s modulus are different in two directions, which shows that the mechanical properties of MoS_2_ sheets are not only affected by porosity but also affected by the shape of the defect hole. In sheets with porosity of 1.56% and 10.56%, the total hole length in the armchair direction is longer than the total hole length in the zigzag direction. So when tensile in the orientation perpendicular to the direction with the larger total hole length, it results in ultimate stress and Young’s modulus are smaller. This result is consistent with previous research that the shape of the pore and the distribution pattern may play an important role^29^.

The failure of the pristine monolayer MoS_2_ membrane under the uniaxial tensile test in the directions of the armchair and zigzag at diverse temperatures is shown as Supplementary Figs. 3, 4. It points out that under uniaxial in armchair direction, temperature has no considerable influence on fracture shape but affects failure strain. The fracture propagates in a path perpendicular to the tensile direction under uniaxial stress along the armchair direction at various temperatures. However, in the zigzag tension, we observed that the temperature affects not only the fracture strain but also the shape and direction of crack propagation. The temperature increases result in reducing the failure strain. At 1 K, the failure strain is 22.791%. At the same time, this value is decreased to 9.488% as the temperature increases to 600 K. In addition, it is observed that at low temperatures (1 K and 100 K), the cracks mainly propagate only in the direction of the black arrow (the zigzag direction). With increasing temperature (200 K to 400 K), the cracks initially propagate in a zigzag direction (black arrows), then expand in a direction perpendicular to the tensile orientation (red arrows). When the temperature is high (500 K and 600 K), the cracks primarily spread perpendicular to the tensile orientation. This phenomenon shows that the temperature affects the crack propagation process; the higher the temperature, the more dominant the cracks propagate in the direction perpendicular to the tensile direction.

**Figure 4:**
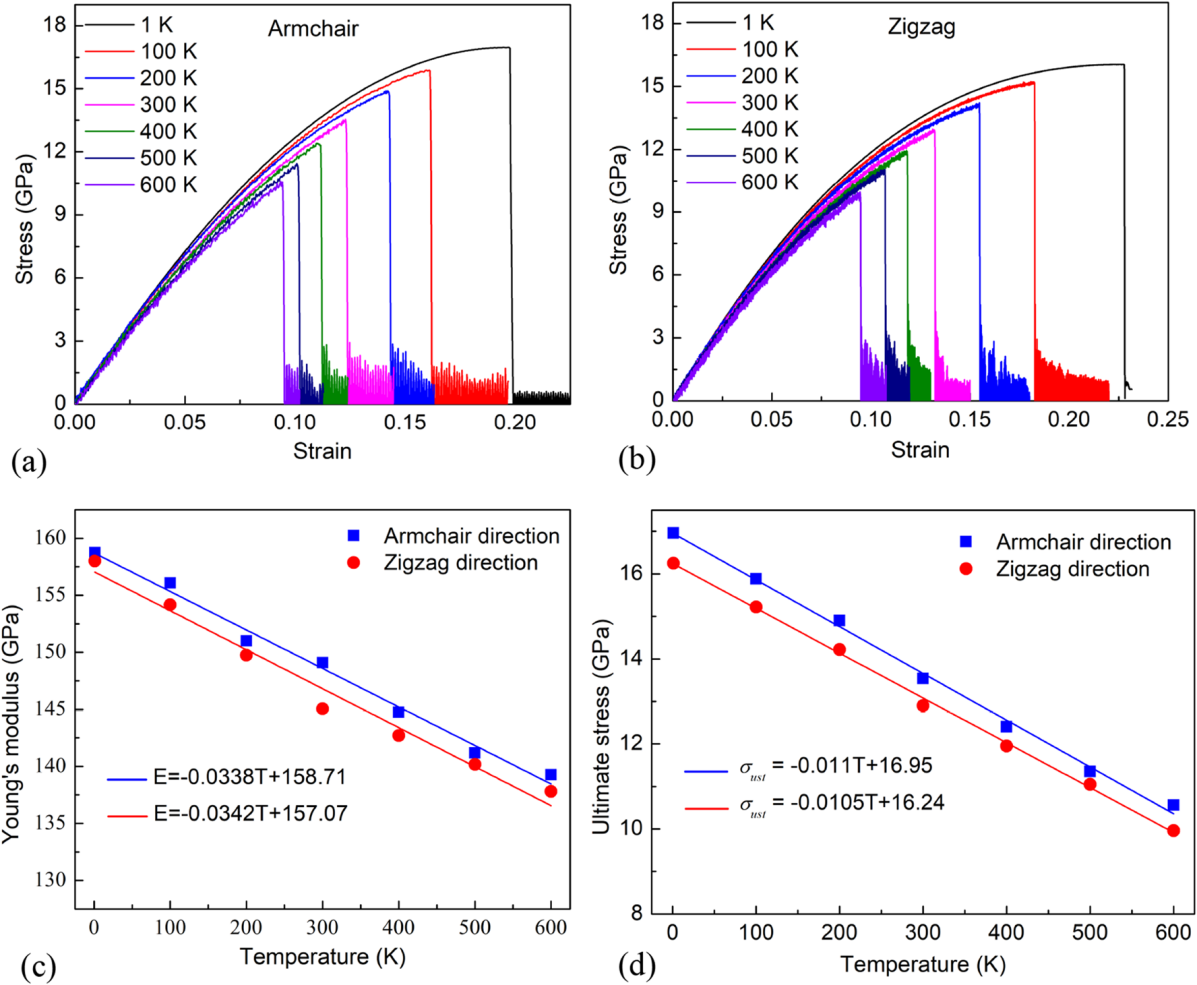
(**a**) Tensile stress–strain curves of monolayer MoS_2_ membrane under tension along the armchair direction with various temperatures. (**b**) Tensile stress–strain curves of monolayer MoS_2_ membrane under tension in the zigzag direction with various temperatures. (**c**) The temperature dependence of Young’s modulus. (**d**) Ultimate stress under uniaxial tension with different temperatures.

The tensile characteristics of the monolayer MoS_2_ membrane at various temperatures are shown in Fig. 4. It is observed that the stress–strain curves are strongly influenced by temperature, where *σ* is the normal stress. The temperature dependences of Young’s modulus and fracture stress of monolayer MoS_2_ membrane are plotted in Fig. 4c,d, respectively. The results demonstrate that the fracture strain, ultimate stress, and Young’s modulus of the membrane decrease with higher temperature. This is explained as follows: higher temperatures will cause stronger thermal oscillations and lead to temperature-induced softening^45^. This strong thermal vibration of the atoms in the sheet makes it easier for chemical bonds to reach critical lengths, and under tensile loads make these bonds susceptible to breakage. The temperature-dependent Young's modulus *E* of the MoS_2_ membrane can be estimated as

1 $$ {\text{For armchair direction}}{:}\; E = - 0.0338T + 158.71\;\left( {{\text{GPa}}} \right) $$

2 $$ {\text{For zigzag direction}}{:}\;E = - 0.0342T + 157.07\;\left( {{\text{GPa}}} \right) $$

From the ultimate stress values of the MoS_2_ monolayer at different temperatures, we can build a linear equation of ultimate stress *σ*_*uts*_ according to the temperature *T* as follows:

3 $$ {\text{For armchair direction}}{:}\;\sigma_{uts} = - 0.011T + 16.95\;\left( {{\text{GPa}}} \right) $$

4 $$ {\text{For zigzag direction}}{:}\; \sigma_{uts} = - 0.0105T + 16.24\;\left( {{\text{GPa}}} \right) $$

The results show that Young's modulus and ultimate stress values under tension loading in the zigzag direction are close to the armchair direction. This result demonstrates that the mechanical properties of the MoS_2_ membrane are nearly isotropic, which is consistent with the earlier study^19,29,46^. The previous studies^46,47^ reported that the mechanical properties of monolayer MoS_2_ are isotropic due to the threefold rotational symmetry in this quasi-hexagonal lattice structure.

**Figure 5:**
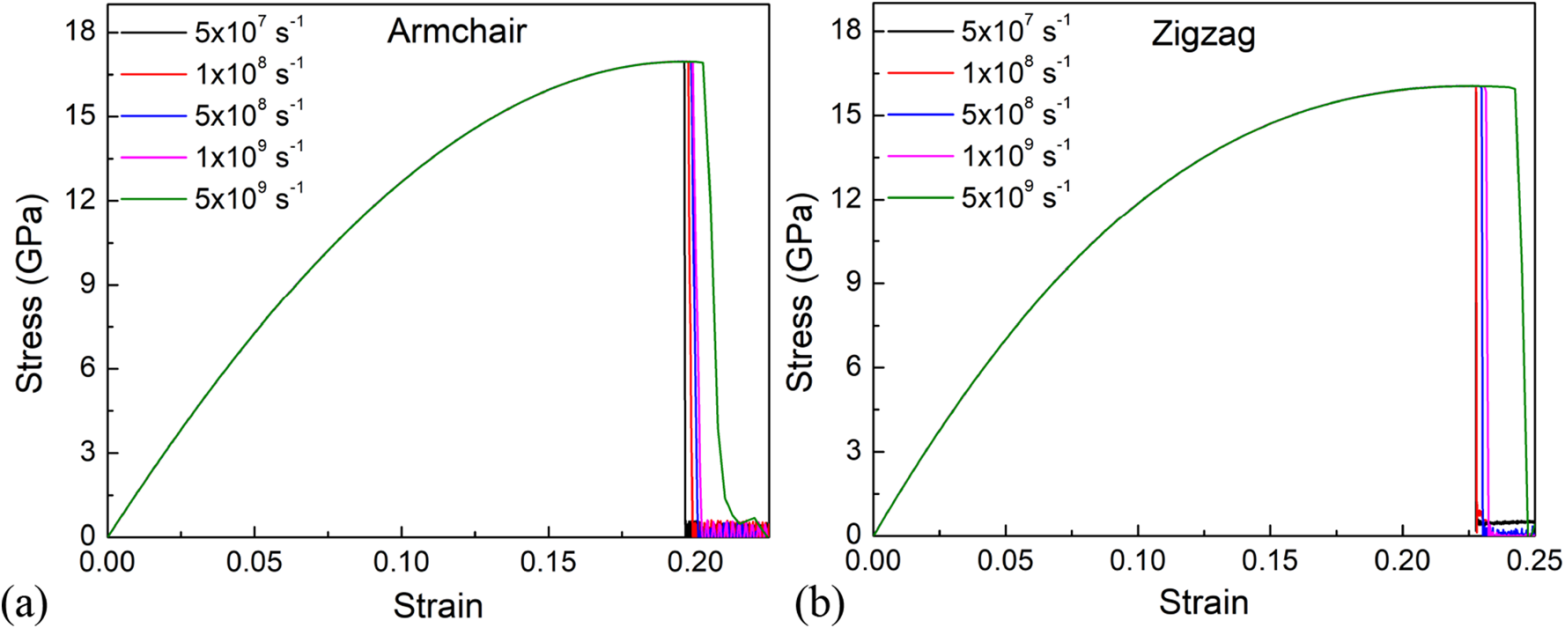
Stress–strain relationship of monolayer MoS_2_ at temperature of 1 K with different strain rates under tension along (**a**) armchair direction, and (**b**) zigzag direction.

To investigate the effect of strain rate on Young's modulus, ultimate stress, and fracture strain, a uniaxially stretched pristine sheet with strain rate varying from 5 × 10^7^ s^−1^ to 5 × 10^9^ s^−1^ is investigated. The tensile stress–strain responses for pristine monolayer MoS_2_ under tension as different strain rates are shown in Fig. 5. The stress–strain curves do not vary with varied strain rates before the fracture occurs, which can be seen. This demonstrates that the strain rate has no effect on Young's modulus of a monolayer MoS_2_ membrane. In addition, as the strain rate increases, the fracture strain and ultimate stress increases but are very small, which is consistent with previous studies for 2D materials. The relaxation period is long enough at a low strain rate to promote bond rearrangement, vacancy coalescence, and crack propagation, resulting in lower fracture stress and fracture strain. At a faster strain rate, the atoms are unable to respond to external forcing in time, resulting in a uniform distribution of broken bonds. As a result, the destructive fracture is slowed, and failure strength is increased^48,49^. However, we can see that the influence of strain rate on the tensile properties of the monolayer MoS_2_ is much smaller than the influence of other factors such as porosity and temperature.

### Biaxial tension

In this section, the biaxial tensile process in both *x* and *y* directions was done concurrently, with the same strain rate as the uniaxial tensile process, in order to evaluate the influence of porosity on the mechanical properties of the MoS_2_ sheet under biaxial tension. We also looked at how porosity and temperature influenced the deformation process, failure morphology, tensile stress, stress–strain relationships, and Young's modulus.

**Figure 6:**
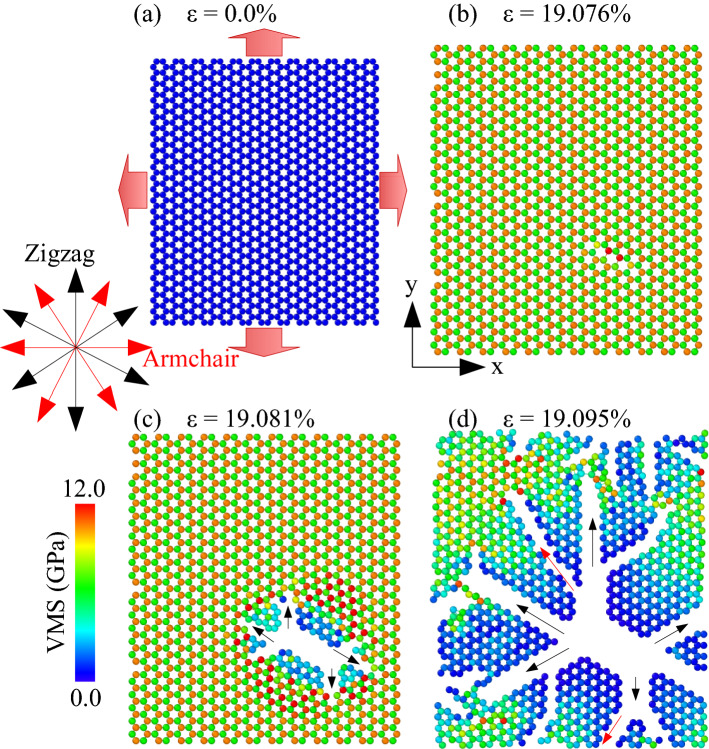
Deformation evolution of single-layer MoS_2_ membrane under biaxial tensile test at 1 K.

Figure 6 reveals the von Mises stress (VMS) evolution and deformation process of the pristine MoS_2_ membrane in the biaxial tensile test at 1 K. The VMS values are used to color all atoms on the membrane; the lower VMS is represented by blue, while the greater VMS is represented by red. The VMS on the membrane grows as the strain increases. The membrane begins to break when the strain gets of 19.076%. As the deformation increases, the cracks rapidly propagate along the black arrow directions (zigzag direction). These cracks rapidly spread in the membrane following the black and red arrows until the membrane is totally destroyed. In general, cracks developed in the zigzag direction (black arrows) were dominant over the armchair direction (red arrows). This demonstrates that the armchair edge has higher binding energy than the zigzag edge. Compared with the uniaxial tensile process, the crack shape of the membrane under biaxial tension is more complex and rough.

Supplementary Fig. 5 shows the VMS distribution and deformation evolution of monolayer MoS_2_ membranes under the biaxial tension at 1 K with different porosities. Each snapshot illustrates the deformation evolution at different strain values, and the atoms are colored according to the VMS value. The results point out that the atoms around the holes will have the highest stress under biaxial tension. As the deformation increases, cracks will begin to appear at these locations. For the sheets with porosity of 1.56%, 4.69%, 10.94%, cracks started to appear when the strain reached the values of 9.950%, 9.834%, 10.111%, respectively. The cracks begin to propagate in the direction of the black arrows. Interestingly, the directions of the black arrows run along the zigzag edge. This demonstrates that the zigzag edge has lower binding energy than the armchair edge. After then, the cracks propagate quickly until the membrane is absolutely wrecked. In biaxial tension, porosity also is not sensitive to the fracture strain.

To survey the effect of temperature on the mechanical properties of monolayer MoS_2_, we performed the biaxial tensile process with the pristine MoS_2_ sheet at temperatures from 1 to 600 K. Supplementary Fig. 6 depicts the VMS distribution and the fracture behavior of monolayer MoS_2_ with various temperatures under biaxial tension. Due to the temperature-induced softening, as the temperature increase, the fracture strain decrease. The black arrows show where the crack started to appear and the direction of its propagation. When the membrane deformation reaches the fracture strain, the cracks also propagate mainly along the black arrows which are also the zigzag edges. This also shows that the binding energy of the zigzag edge is lower than that of the armchair edge. At high temperatures (500 K and 600 K) cracks perpendicular to the y-direction also begin to appear (the red arrows—armchair edge), but much less than that in uniaxial tension. To further study the effect of temperature on the change of crack shape under biaxial tension, we carried out the survey with higher temperatures (800 K, 900 K, 1000 K). The results show that the higher the temperature, the more cracks appear in the direction perpendicular to the tensile direction. Especially under biaxial tension with a temperature of 900 K or more, the cracks in the direction perpendicular to the tensile direction are more dominant, as shown in Supplementary Fig. 6g–k. In addition, the results show that under the biaxial tensile process, the crack shape of the membrane is more complicated, the membrane morphology is rougher than that under uniaxial tension. Furthermore, the results demonstrate that the failure strain in the biaxial test is less than the failure strain in the uniaxial tensile test at each temperature range. It indicates that the MoS_2_ membrane is more vulnerable to destruction under biaxial tension than under uniaxial tension.

**Figure 7:**
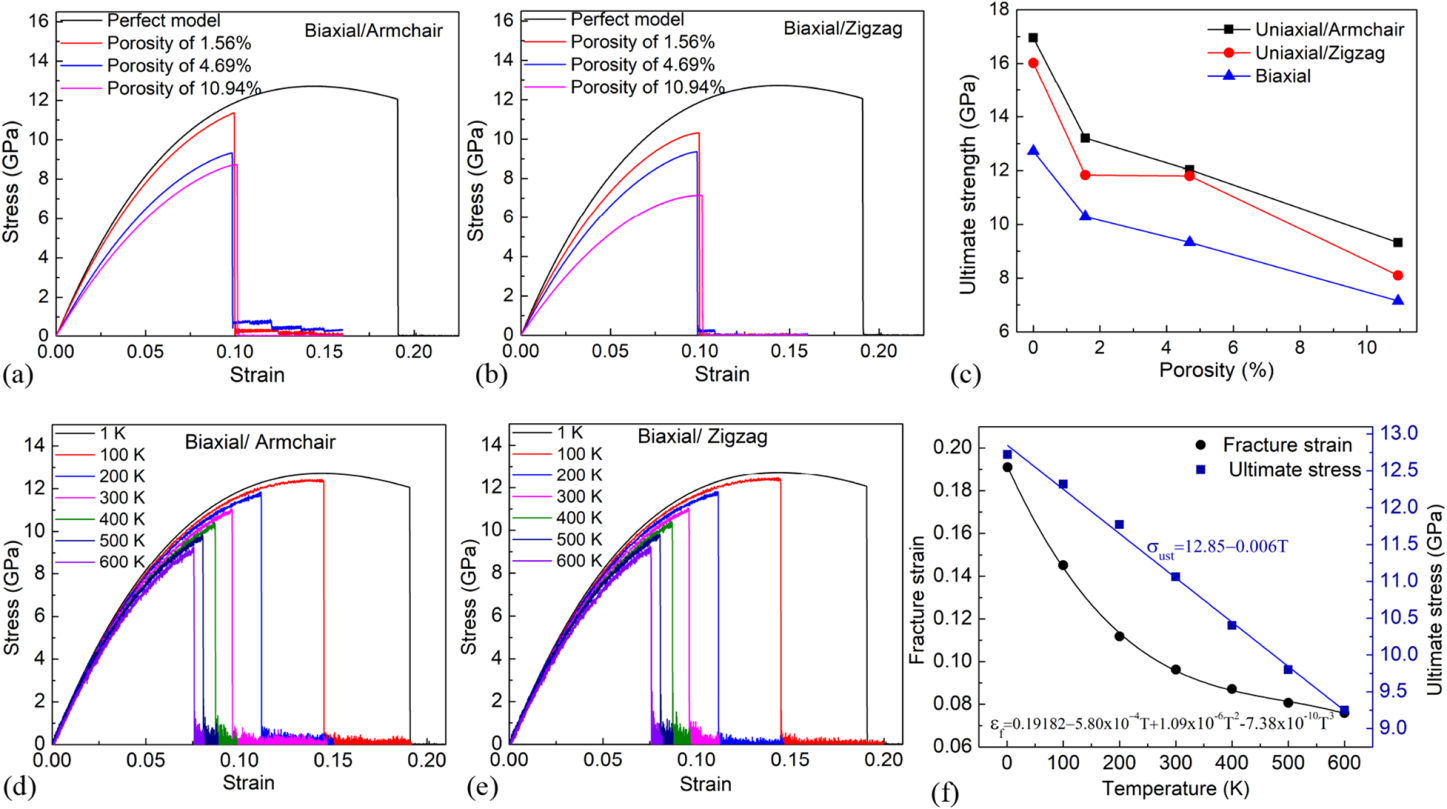
(**a**) Tensile stress–strain curves of monolayer MoS_2_ membrane in the armchair direction under biaxial tension at 1 K with various porosities. (**b**) Tensile stress–strain curves of monolayer MoS_2_ membrane in the zigzag direction under biaxial tension with various porosities. (**c**) The porosity dependence of ultimate strength. (**d**) Tensile stress–strain curves of monolayer MoS_2_ membrane in the armchair direction under biaxial tensile test with different temperatures. (**e**) Tensile stress–strain curves of monolayer MoS_2_ membrane in the zigzag direction under biaxial tensile test with different temperatures. (**f**) The temperature dependence of ultimate stress and fracture strain.

Figure 7a–c depicts the tensile characteristics of the MoS_2_ membranes with diverse porosities under the biaxial tension at 1 K. The stress–strain curves of single-layer MoS_2_ membranes in the armchair and zigzag directions are plotted from the biaxial tensile test data, as presented in Fig. 7a,b, respectively. The results demonstrate that porosity has a substantial impact on the material’s mechanical characteristics. As porosity increases, ultimate strength decreases. Specifically, under the biaxial tension at 1 K, the ultimate strength of the membranes with various porosities of 0%, 1.56%, 4.69%, 10.94% has values of 12.72, 10.29, 9.33, and 7.15 GPa, respectively. When the membranes have holes, the fracture strain is greatly reduced compared to the pristine membrane; however, with increasing porosity, the fracture strain is insensitive to porosity. The ultimate strength values of the MoS_2_ membranes under the uniaxial tensile along the zigzag and armchair directions are shown in Fig. 7c for convenience of comparison in biaxial and uniaxial tensile. Compared with the uniaxial tensile test, the ultimate strength of the membrane in the biaxial tensile test is smaller. The mechanical properties of the pristine monolayer MoS_2_ under biaxial tension at different temperatures are shown in Fig. 7d–f. It points out that temperature has a remarkable impact on the stress–strain relationship. The temperature-induced softening lead to mechanical properties reducing as the temperature increases. The results point out that a much lower ultimate stress and fracture strain are observed as the temperature grows. Moreover, the results we survey with various temperatures reveal that under biaxial tension, the ultimate strength of the single-layer MoS_2_ sheet is lower than that of uniaxial tension. The relationship between temperature *T* and ultimate stress *σ*_*uts*_ can be described by the linear fitting equation follow:

5 $$ \sigma_{uts} = - 0.006T + 12.85\;({\text{GPa}}) $$

The relationship between temperature and fracture strain *ε*_*f*_ can be described by the equation follow:

6 $$ \varepsilon_{f} = 0.19182 - 5.8 \times 10^{ - 4} T + 1.09 \times 10^{ - 6} T^{2} - 7.38 \times 10^{ - 10} T^{3} $$

### Thermal conductivity

This section investigated the effects of porosity and temperature on the thermal conductivity of nanoporous MoS_2_ membranes. Calculating the thermal conductivity at infinite length (intrinsic thermal conductivity) requires two steps: after a series of size-dependent simulations, a size-independent extrapolation is performed. Therefore, for each porosity and temperature to be investigated, we will simulate with different lengths to calculate the intrinsic thermal conductivity value.

**Figure 8:**
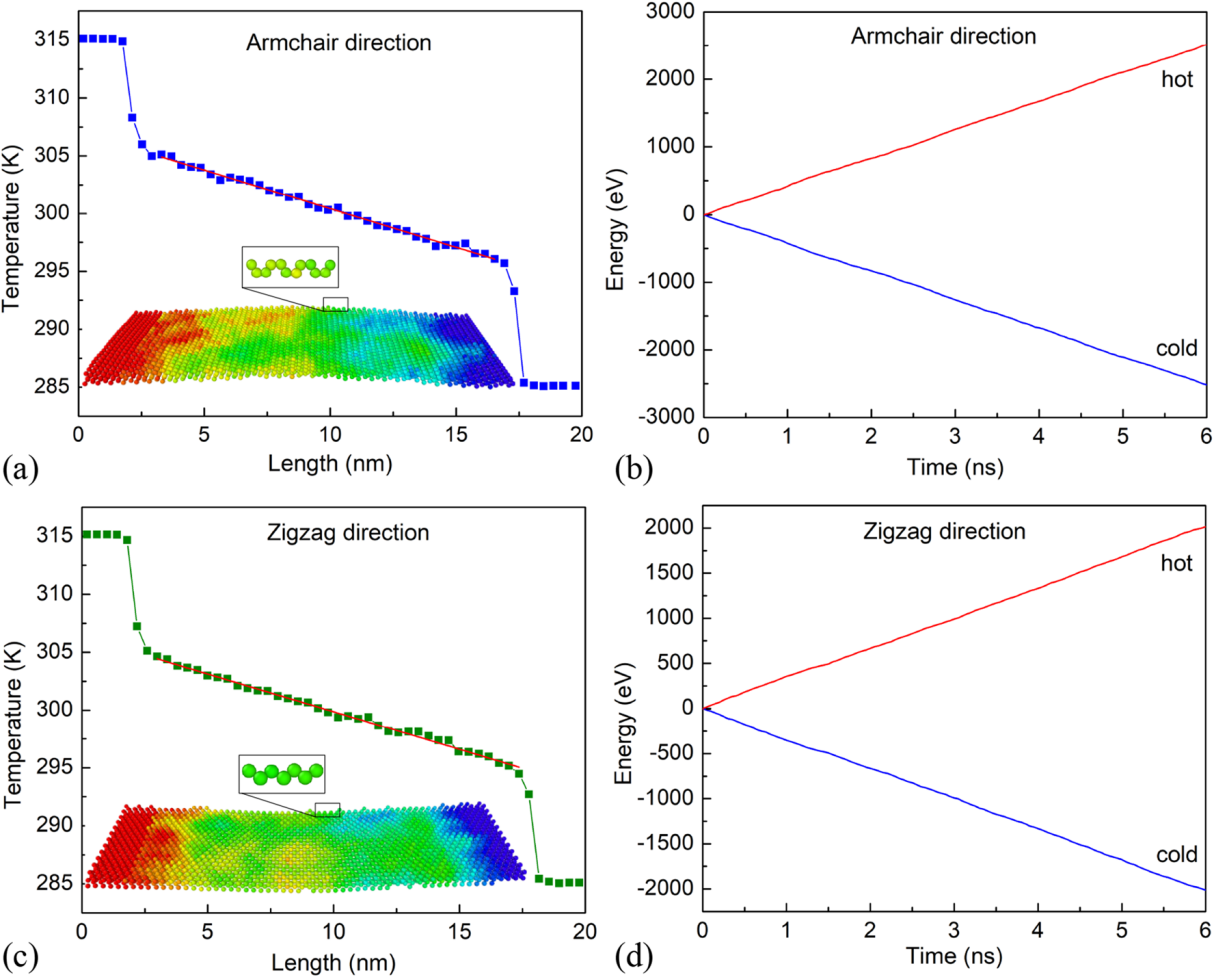
(**a**) Temperature distribution along the armchair direction of *L* = 19.4 nm with the ambient temperature of 300 K. (**b**) Energy change in the cold and hot zones with respect to the time during heat flow transfer along the armchair direction. (**c**) Temperature distribution along the zigzag direction of *L* = 19.96 nm with the ambient temperature of 300 K. (**d**) Energy change in the cold and hot zones according to the time during heat flow transfer along the zigzag direction.

A typical temperature profile at a steady state is shown in Fig. 8. Figure 8a,c illustrates the temperature distribution on the membrane in the directions of armchair and zigzag at 300 K with *L* = 19.4 nm, and *L* = 19.96 nm, respectively. The color distribution in the sheet represents the temperature of each atom. Temperatures are highest in the hot region and lowest in the cold region. The temperature gradually decreases from the hot zone to the cold zone. At steady-state conditions, the temperature gradient profile (*dT/dx*) depicts linear behavior as the red line. Furthermore, we obtained the energy supplied to the hot zone and subtracted from the cold zone over simulation time, as plotted in Fig. 8b,d. Where the red line denotes the energy added to the hot zone, and the blue line is the energy subtracted from the cold region. We observed that the energy added to the hot zone and subtracted from the cold zone is linear in time. Moreover, the energy subtracted from the cold region is equal to the energy supplied to the hot region, representing conservation of energy and a constant heat-flux, was imposed on the system. From these linear lines, we can calculate *dE/dt/A* (the average heat flux along the gradient).

**Figure 9:**
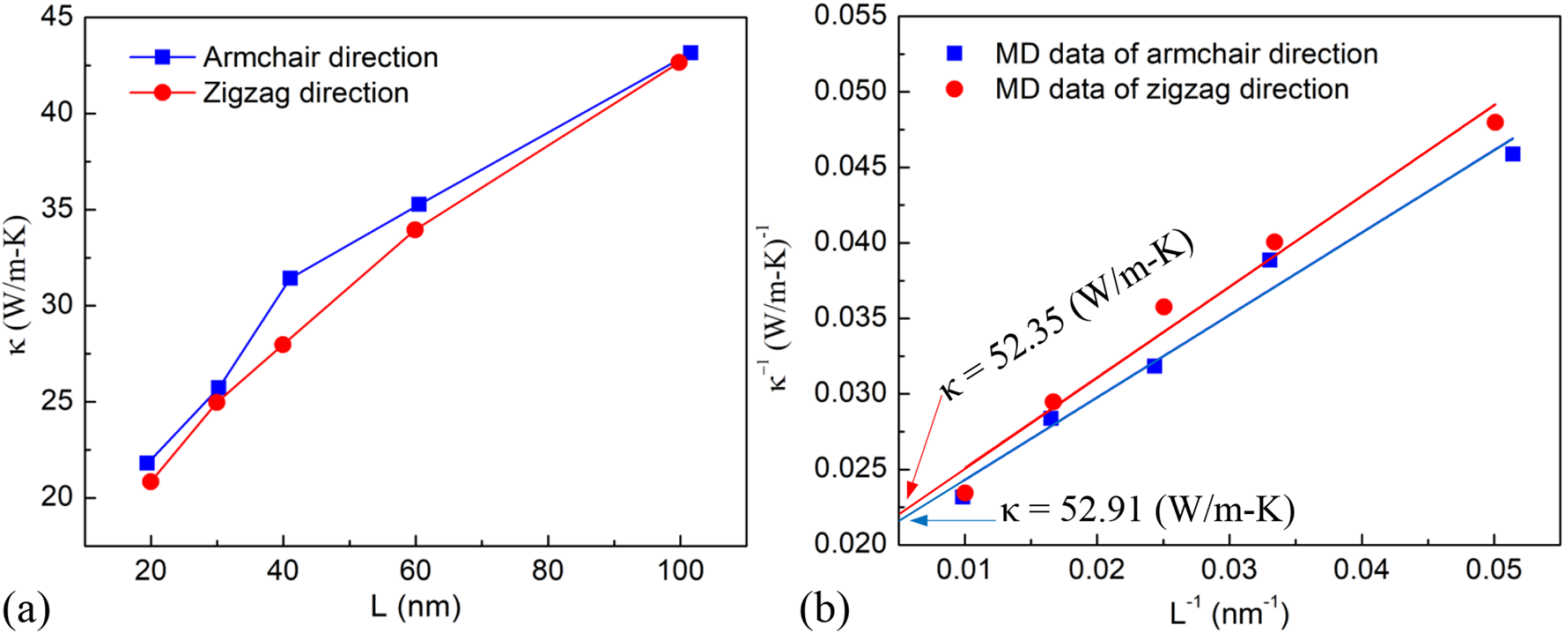
(**a**) The relationship between the thermal conductivity and the length of the monolayer MoS_2_ membrane in the armchair and zigzag directions at an ambient temperature of 300 K. (**b**) The linear fitting results of *1/L* and *1/κ* of monolayer MoS_2_ membrane in the armchair and zigzag directions.

Based on the established temperature gradient and the applied heat-flux, applying formula (20), we can calculate the thermal conductivity for the MoS_2_ membrane with a length of 19.45 nm in the armchair direction to be 21.79 (W/m K) and 20.84 (W/m K) in zigzag direction with a length of 19.96 nm. Similarly, for the variable lengths from 30.2 to 101.5 nm, we calculate the *κ* values to be 25.73, 31.42, 35.26, and 43.15 in the armchair direction. Besides, the *κ* values were calculated to be 24.97, 28.97, 33.94, and 42.65 in the zigzag direction. These values are plotted in Fig. 9a. The results show that the sample size has a great influence on thermal conductivity. As the sample length increases, the value of *κ* increases. The *κ* rises with rising sample length because of the decrease of phonon-boundary scattering. To obtain the intrinsic thermal conductivity of monolayer MoS_2_, we use the extrapolation of the NEMD results for the samples with different lengths. The extrapolation formula is mentioned by Schelling et al.^50^.

7 $$ \frac{1}{\kappa (L)} = \frac{1}{{\kappa_{\infty } }}\left( {\frac{\lambda }{L} + 1} \right) $$

here, *L* represent the sample length, *κ*_*∞*_ denotes the intrinsic thermal conductivity of an infinitely long sample, and *λ* is the effective phonon mean free path.

From the linear fitting of *1/L* and *1/κ* in Fig. 9b, the intrinsic thermal conductivity of perfect MoS_2_ membrane can be obtained by extrapolating at the infinitely long sample (*1/L* → 0), and it was be found to be 52.91 W/m K and 52.35 W/m K in the directions of the armchair and zigzag, respectively. These results are in line with the previous studies^51^. It is consistent with an experimental study that has estimated that few-layer MoS_2_ has a thermal conductivity of 52 W/m K^52^. Other experimental measurements showed thermal conductivity of monolayer MoS_2_ *κ* = (34.5 ± 4) W/m K at room temperature^32^, *κ* = (48–52) W/m K at room temperature^53^. The effective phonon mean free path in the armchair and zigzag directions at 300 K were found 29.8 nm and 31.4 nm, respectively. Interestingly, the values of *κ* in the armchair direction are considerably near to the zigzag direction.

**Figure 10:**
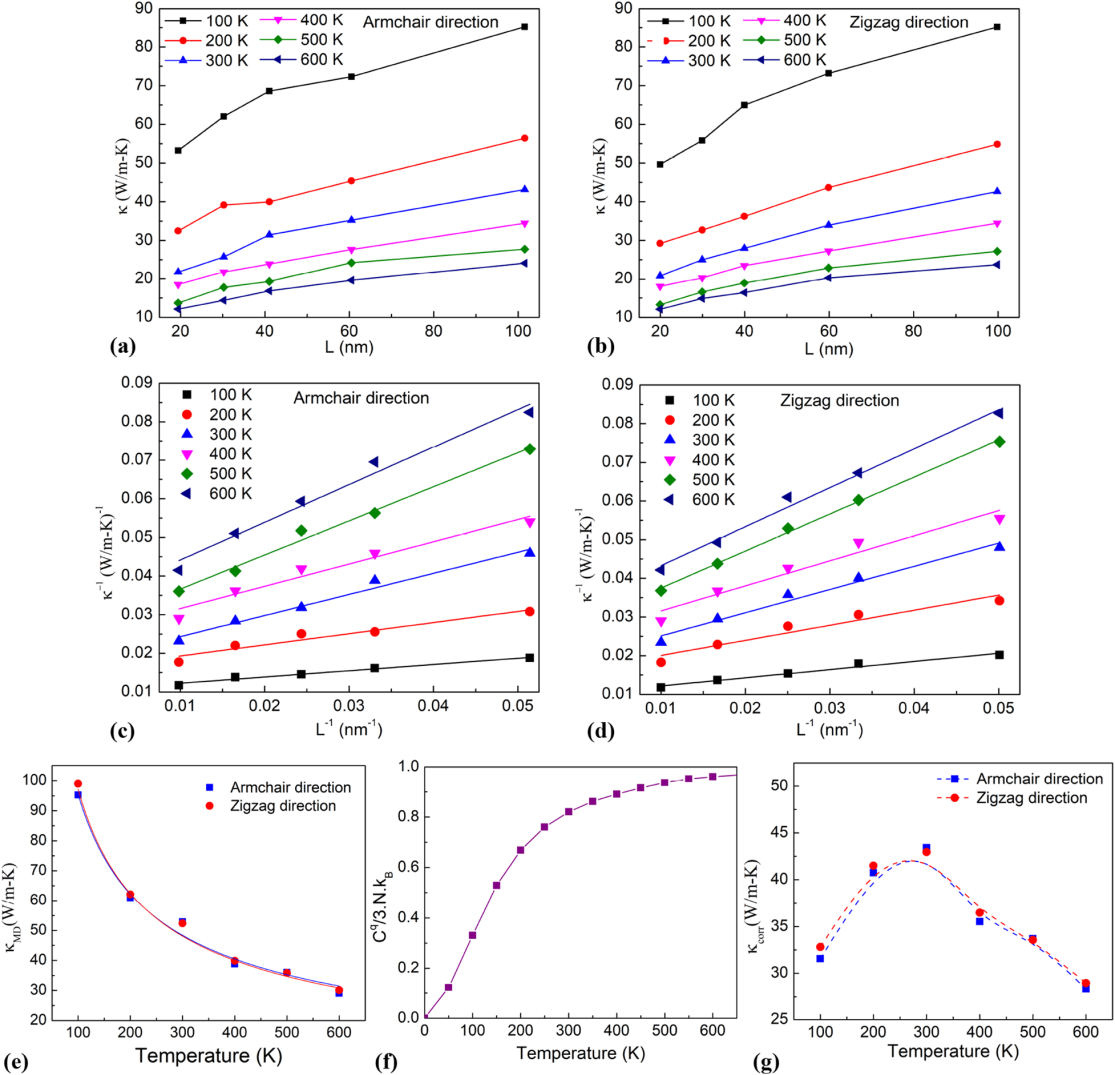
(**a**,**b**) The relation between the thermal conductivity and the length of the monolayer MoS_2_ membrane in the armchair and zigzag directions at various temperatures. (**c**,**d**) The linear fitting results of *1/L* and *1/κ* of monolayer MoS_2_ membrane in the armchair and zigzag directions at various temperatures. (**e**) NEMD-calculated thermal conductivity. (**f**) Computed temperature-dependent specific heat *C*^*q*^. (**g**) The quantum-corrected thermal conductivity.

Figure 10a,b illustrates the relation between the thermal conductivity and the length of the monolayer MoS_2_ membrane in the armchair and zigzag directions at different temperatures. It indicates that as the temperature rises, the thermal conductivity decreases with the same sample length. In recent studies on MoS_2_ materials^30,51,54^, it has been explained that the thermal conductivity reduces as growing temperature due to the stronger phonon–phonon scattering at higher temperatures which reduces the phonon mean free path (MFP). Specifically, more phonons are excited with increasing temperature, resulting in more phonon–phonon scattering and reduced phonon MFP. The thermal conductivity is proportional to the phonon MFP, according to kinetic theory. As a result, as the temperature rises, the thermal conductivity drops. Similar temperature dependence is also found in other 2D materials such as graphene, MoSe_2_, h-BN^55–57^. In addition, at each temperature, the thermal conductivity increases as the sample length increases. The relation between *1/κ* and *1/L* of monolayer MoS_2_ with different sample lengths at various temperatures in the armchair and zigzag directions are plotted in Fig. 10c,d. The intrinsic thermal conductivity at various temperatures was obtained by the extrapolation value when *1/L* → 0. The extrapolated conductivity values at various temperatures by MD simulations are plotted in Fig. 10e. It is worthy to note that the thermal conductivity decrease with increasing temperature. This trend is consistent with our previous studies^58,59^ that have been studied for other 2D materials. This can be explained as follows: based on the principle of thermal conductivity^60^, the thermal conductivity can be expressed by the following expression:

8 $$ \kappa = \frac{1}{3}Cvl $$

here, *C* denotes the specific heat per volume, *l* represents the phonon mean free path, *v* is the average phonon velocity.

For the perfect materials, the thermal conductivity is mostly controlled by the phonon–phonon scattering^61^:

9 $$ \kappa = \frac{{\kappa_{B}^{2} \theta_{D} }}{{2\pi^{2} v\hbar CT}} $$

where $\hbar$ is the Planck constant, $\kappa_{B}$ is the Boltzmann constant, $\theta_{D}$ is the Debye temperature.

As a result of phonon–phonon scattering, thermal conductivity is inversely proportional to temperature, that is $\kappa \propto 1/T$. Therefore, our calculation results are in accordance with the principle of thermal transport calculation. However, the thermal conductivity decreases as increasing the temperature, which is compatible with experimental measurements only at a high temperature above the Debye temperature, *θ*_*D*_^62^. Due to MD having the significant limitation of being entirely classical, with each vibrational mode equally excited; thus, it is only rigorously applicable to solids above the Debye temperature. Peng et al.^63^ calculated the Debye temperature of MoS_2_ to be 262.3 K. Thereby, calculated *κ*_*MD*_ values for T > θ_D_ > 262.3 K are realistic. Below Debye temperature (T < 262.3 K), the quantization of vibrational energy becomes the major cause of inaccuracy in NEMD-calculated thermal conductivity. The specific heat remains constant *C*^*MD*^ = *3Nk*_*B*_ at all temperatures in classical NEMD simulations. However, for temperatures below the Debye temperature, quantum effects become important, and the experimentally realistic value of *C*^*q*^*(T)* decreases. Therefore, the corrections for $\kappa$ as low temperatures have been mentioned in^51^.

10 $$ \kappa_{corr} = \kappa_{MD} \times \frac{{C^{q} (T)}}{{C^{MD} }} $$

From the equation describes the crystal's quantized vibrational energy:

11 $$ \left\langle E \right\rangle = \int {\hbar \omega BE(\omega ,T)G(\omega )d\omega } $$

here, $BE(\omega ,T) = \left( {\exp \left( {\frac{\hbar \omega }{{\kappa_{B} T}}} \right) - 1} \right)^{ - 1}$ is the temperature-dependent Bose–Einstein distribution. *G(ω)* denotes the phonon density of state, and *ω* is the phonon frequency. Therefore, the lattice specific heat *C*^*q*^*(T)* is given by:

12 $$ C^{q} \left( T \right) = \frac{d\left\langle E \right\rangle }{{dT}} = \kappa_{B} \int {\left( {\frac{\hbar \omega }{{\kappa_{B} T}}} \right)^{2} } \frac{{\exp \left( {\frac{\hbar \omega }{{\kappa_{B} T}}} \right)}}{{\left( {\exp \left( {\frac{\hbar \omega }{{\kappa_{B} T}}} \right) - 1} \right)^{2} }}G\left( \omega \right)d\omega $$

13 $$ {\text{Thus}},\;\;\frac{{C^{q} }}{{3N\kappa_{B} }} = \frac{{\int\limits_{0}^{\infty } {\frac{{u^{2} e^{u} }}{{\left( {e^{u} - 1} \right)^{2} }}G\left( \omega \right)d\omega } }}{{\int\limits_{0}^{\infty } {G\left( \omega \right)d\omega } }} $$

here $$u = \frac{\hbar \omega }{{k_{B} t}}.$$

Figure 10f depicts the computed (*C*^*q*^*/C*^*MD*^), and Fig. 10g shows the quantum-corrected *κ*_*corr*_ of the monolayer MoS_2_ membrane at different temperatures. It was found that the quantum-corrected *κ*_*corr*_ of monolayer MoS_2_ membrane at 300 K to be 43.41 W/m K and 42.92 W/m K in the directions of the armchair and zigzag, respectively. Moreover, the results show that the thermal conductivity values of the MoS_2_ monolayer in the zigzag direction and the armchair direction are very similar.

**Figure 11:**
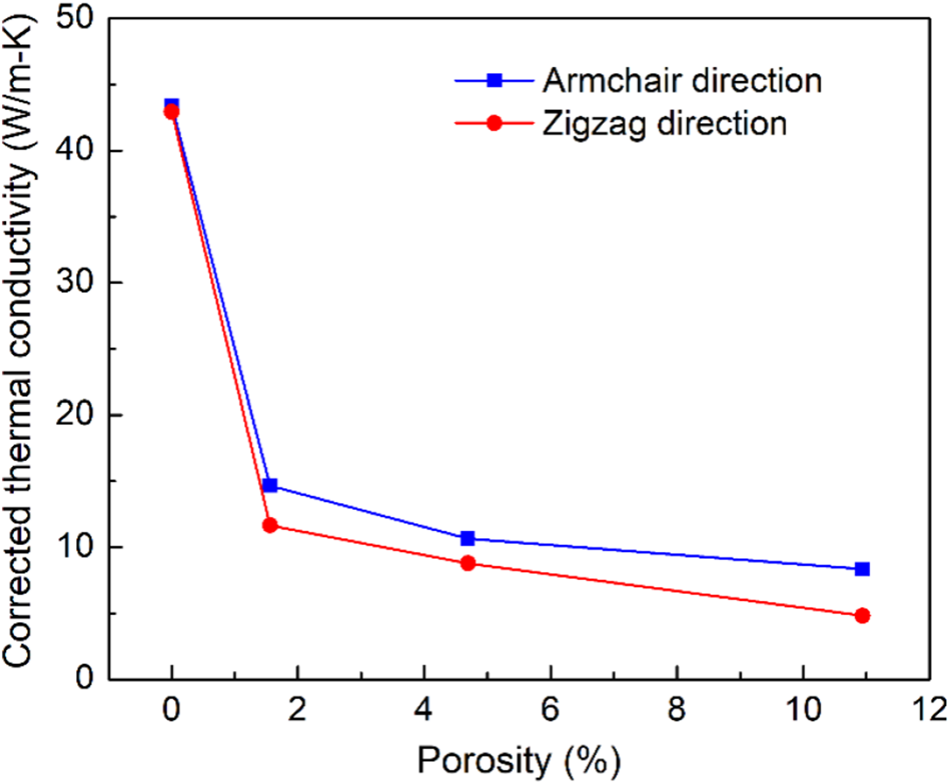
Porosity dependence of the intrinsic thermal conductivity of single-layer MoS_2_ membrane.

The NEMD thermal conductivity dependence on the sample length of single-layer MoS_2_ membrane in the armchair and zigzag directions for various porosities is displayed in Supplementary Fig. 7a,b. It points out that the porosity strongly affects on thermal conductivity of the material. With the same sample length, as the porosity increases, the thermal conductivity decreases. Supplementary Fig. 7c,d displays the relation between *1/L* and *1/κ* for armchair and zigzag directions with various porosities. The intrinsic thermal conductivities at various porosities were obtained by the extrapolation value when *1/L* → 0. The extrapolated thermal conductivity values at various porosities are corrected and plotted in Fig. 11.

It can be clearly seen that the thermal conductivity reduces as increasing the porosity density, as shown in Fig. 11. These significant reductions of the thermal conductivities can be mainly attributed to the phonon-defect scattering. According to the thermal transport principle^60^, different defects scatter lattice waves and decrease the phonon mean free path. With the porous system, the effective mean free path is altered to^41,42,60^:

14 $$ \frac{1}{{l_{eff} }} \propto \frac{1}{{l_{phonon - phonon} }} + \frac{1}{{l_{phonon - defect} }} $$

here, *l*_*phonon–phonon*_ represents the phonon–phonon scattering length, and *l*_*phonon–defect*_ denotes the scattering length due to defects.

By the Eqs. (8) and (14), the *κ* of defective material meets the relationship:

15 $$ \frac{1}{\kappa } \propto \frac{1}{{l_{phonon - phonon} }} + \frac{1}{{l_{phonon - defect} }} $$

As previously studied^41,42,60^, the phonon-defect scattering raises as the defect density rises, leading to the reduction of *l*_*phonon–defect*_. Consequently, from Eq. (15), it is indicated that the thermal conductivity of monolayer MoS_2_ reduces with rising porosity because of the phonon scattering induced by the defects. We can see that these MD simulation results in this study are consistent with previous studies^54,64,65^.

To understand the mechanism responsible for the decrease in thermal conductivity of defective membrane, the phonon density of states (DOS) is used to explain this mechanism. The DOS is calculated using the Fourier transform of atomic velocities autocorrelation function at an equilibrium state^57^:

16 $$ G\left( \omega \right) = \int {\left\langle {\sum\limits_{i = 1}^{N} {v_{i} (t)v_{i} (0)} } \right\rangle } e^{ - i\omega t} dt $$

where *N* denotes the number of atoms in the system, and *v*_*i*_*(t)* is the velocity of atom *i*-th at time *t*. $\left\langle {...} \right\rangle$ denotes atom number-averaged velocity autocorrelation function.

**Figure 12:**
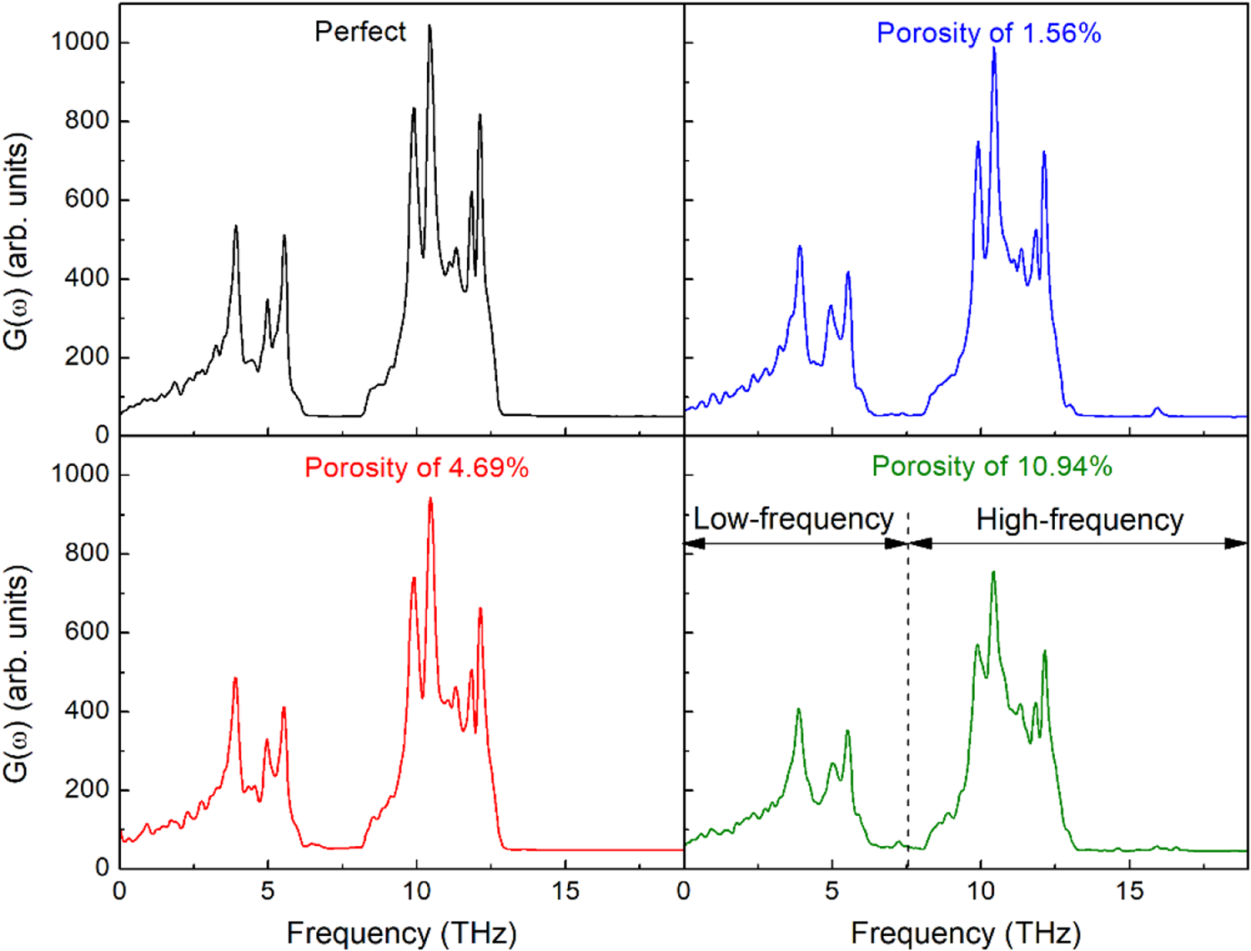
Calculated vibrational density of states for single-layer MoS_2_ membranes.

The MoS_2_ monolayers of size 7.48 × 19.4 (*W*×*L*) nm^2^ with different porosities are performed for the calculation of velocity autocorrelation function at a temperature of 300 K. Figure 12 illustrates the DOS of MoS_2_ monolayers with different porosities at 300 K. As can be seen from the figure that the porosity has a significant effect on the DOS. The DOS peaks in both the low-frequency band (0–7.5 THz) and high-frequency band (7.5–20.0 THz) of nanoporous membranes are reduced, and most of the peaks are widened compared to those of pristine monolayer MoS_2_ membrane. The more porosity increases, the more DOS peaks are attenuated. These changes reflect the scattering impact of defects on phonons, which reduces phonon life and reduces thermal conductivity significantly^66^.

We compare the results of the perfect model with previous studies to validate the computational model. The comparison of the calculated results with some relevant literature is shown in Supplementary Table 1. Despite multiple investigations of monolayer MoS_2_ thermal conductivity, considerable discrepancies in thermal conductivity values were discovered owing to variances in techniques, force fields, and so on^54^. Supplementary Table 1 demonstrates that our results are consistent with previous DFT calculations^35,38^, experiments^31,32,52,53^, and MD simulations^29,51,54^. Therefore, the results of our study on the influence of temperature and porosity on mechanical properties and thermal conductivity of single-layer MoS_2_ membrane are useful knowledge and can be provided for next researches about MoS_2_ in the future.

## Discussion

In summary, MD simulations were performed to explore the effects of temperature and porosity on the mechanical characteristics of nanoporous two-dimensional MoS_2_ membranes in the uniaxial tensile test and biaxial tensile test. The MoS_2_ membranes with various porosities of 0.0%, 1.56%, 4.69%, and 10.94% were conducted in this investigation. It is found that the temperature significantly affects the mechanical characteristics of the MoS_2_ membranes. With higher temperature, the ultimate stress, fracture strain, and Young modulus of the membrane reduce. The results show that Young's modulus and ultimate stress values in the armchair direction are close to the zigzag direction under the same testing conditions. This result proves that the MoS_2_ membrane is nearly isotropic in mechanical characteristics. Furthermore, results depict an overall reducing trend in Young’s modulus and tensile strength as porosity increases. With the nanoporous membranes, due to the existence of holes, stress is highly concentrated around the hole position in tension, leading as the strain increases, the atomic bonds at these positions will break first. Also, due to stress concentration at these positions, the ultimate strength and failure strain of the porous sheets are reduced from that of the pristine sheet.

We also study the influences of temperature and porosity on the thermal conductivity of monolayer MoS_2_ membranes via NEMD simulations. The length effect on the thermal conductivity of nanoporous two-dimensional MoS_2_ membranes has been studied. It was shown that the thermal conductivity increases with increasing the system size. The intrinsic thermal conductivities of monolayer MoS_2_ have predicted 43.41 W/m K and 42.92 W/m K in the directions of armchair and zigzag at 300 K. It is worthy to note that the thermal conductivity reduces with raising the temperature above the Debye temperature. Moreover, the thermal conductivity of the monolayer MoS_2_ membrane at low temperature was corrected due to the suppression of specific heat. The NEMD results illustrated that the porosity significantly affects thermal conductivity. It is found that the thermal conductivity decreases as the porosity increases due to the phonon scattering induced by the defects. Furthermore, the results reveal that the thermal conductivity of the MoS_2_ membrane in the armchair direction is similar in the zigzag direction.

## Method

### MD simulations for the tension process

A model of porous monolayer MoS_2_ with various porosities was utilized for the biaxial and uniaxial tensions, as presented in Fig. 1a, having *x* and *y* dimensions of 6.48 nm and 7.48 nm, respectively. Furthermore, a 5.0 nm vacuum gap is created above and below the MoS_2_ nanosheet in the *z*-direction to prevent interactions between neighbouring sheets. Periodic boundary conditions are implemented in all dimensions to avoid the influence of the simulation box boundary. Uniaxial and biaxial tensile procedures were used to investigate the mechanical properties of nanoporous two-dimensional MoS_2_ membranes. The system has an equilibrium process prior to the tension test that uses the conjugate gradient (CG) method to achieve an equilibrium minimum energy. Afterwards, the system is relaxed at predefined temperatures using the isothermal and isobaric (NPT) ensemble for 250 ps with a simulation time step of 0.5 fs. Thereafter, the uniaxial or biaxial tension is applied with a constant strain rate of 10^8^ s^−1^, which is typically employed in MD simulations. The tension loading is applied in the NPT ensemble using a simulation time step of 0.5 fs. A Stillinger–Weber potential is built by Jiang et al.^46^ was utilized to describe the interatomic interactions in the MoS_2_ membrane.

To evaluate the mechanical properties of the nanoporous two-dimensional MoS_2_ membranes, the internal stress was monitored during the tensile loading. At the atomic level, the stress component can be defined by the virial theorem^67^

17 $$ \sigma = \frac{1}{V}\sum\limits_{a \in V} {\left[ { - m_{a} v_{a} \otimes v_{a} + \frac{1}{2}\sum\limits_{a \ne b} {(r_{ab} \otimes F_{ab} )} } \right]} $$

here, *v*_*a*_ and *m*_*a*_ represent the velocity vector and the mass of the atom *a*. *V* is the volume of the structure with a thickness of 0.615 nm. *r*_*ab*_ is the distance vector between particle *a* and particle *b*. The symbol $\otimes$ denotes the tensor product of two vectors. *F*_*ab*_ is the force vector between particles *a* and *b*.

The von Mises stress *σ*_*von*_ is given by:

18 $$ \sigma_{von}^{2} = \frac{1}{2}\left[ {\left( {\sigma_{xx} - \sigma_{yy} } \right)^{2} + \left( {\sigma_{yy} - \sigma_{zz} } \right)^{2} + \left( {\sigma_{zz} - \sigma_{xx} } \right)^{2} + 6\left( {\sigma_{xy}^{2} + \sigma_{yz}^{2} + \sigma_{zx}^{2} } \right)} \right] $$

### Thermal conductivity calculations

The schematic illustration of the NEMD thermal conductivity calculations of the monolayer MoS_2_ membrane is displayed in Fig. 1b. The dimension of the monolayer MoS_2_ sample is *W × L*, with *W* and *L* are the width of the sample and the dimension of the heat transfer direction, respectively. The porosity of the sample is the same as the model in Fig. 1a. To eliminate the width dimension's boundary effect on the result, in the width direction of the model, a periodic boundary condition is utilized, while the free boundary condition is utilized in the *z* (out-of-plane) direction^68^. The thermal conductivity values obtained from the NEMD method are significantly affected by the length of samples^41,42^. Thus, the width of the chosen sample is *W* = 7.48 nm, while *L* is varied in the range of 19.4, 30.2, 41.0, 60.5, and 101.5 nm in the armchair direction, and 19.96, 29.94, 39.92, 59.88, 99.80 nm in the zigzag direction. The length size along the two directions, zigzag and armchair, has a small deviation due to the lattice constants *a*_*1*_ and *a*_*2*_ in the rectangular unit cell being different.

Prior to executing the heat flux process, NVT and NVE methods were used to equilibrate the initial configuration at a given temperature in 500 ps intervals, with a time step of 0.5 fs. After obtaining the equilibrate structure, we divided the simulation box along the heat flux direction into 50 slabs to evaluate the local temperature. The cold and hot regions were controlled at *T*_*c*_ = *T(1* − *λ)* K and *T*_*h*_ = *T(1* + *λ)* K by Langevin thermostats, where *λ* = 0.05, and *T* varied from 100 to 600 K. There is a region between the cold and hot zones which is called the heat-conducting region. The heat-conducting region was not connected to any thermostats or thermal reservoir, and it was applied the constant volume and constant energy (NVE) ensemble. Therefore, constant heat flux was imposed in the system by the energy was continuously added to the hot zone and removed from the cold zone. The NEMD simulations were performed for a period of 6.0 ns (12 × 10^6^ time steps). The heat flux can be determined as follows^69^:

19 $$ J = \frac{dE/dt}{A}. $$

here *A* is the cross-sectional area of the MoS_2_ sheet; we chose the thickness of monolayer MoS_2_ membrane of 0.615 nm; this is the experimentally measured thickness for a layer of MoS_2_^70,71^, which was consistent with previous studies^72,73^. *dE/dt* stands for the average heat flux along the gradient. After a transient duration, the temperature gradient (*dT/dx*) along the sample length was determined using the time-average temperature profile. Finally, the thermal conductivity was determined by Fourier’s law:

20 $$ k = \frac{J}{dT/dx}. $$

All MD simulations were performed by LAMMPS software^74^. A Stillinger–Weber potential is built by Jiang et al.^46^ was utilized to model the atomic interactions in the MoS_2_ membrane. This potential has been widely employed in prior works for molecular dynamics simulation of MoS_2_ sheets^26,29,54,75,76^. We used OVITO software^77^ to visualize and analyze the simulation results.

## Supplementary Information

Supplementary Figure 1.

Supplementary Figure 2.

Supplementary Figure 3.

Supplementary Figure 4.

Supplementary Figure 5.

Supplementary Figure 6.

Supplementary Figure 7.

Supplementary Table 1.
