# Supplementary material for: Thermal and mechanical characterization of nanoporous two-dimensional MoS2 membranes
Source: Sci Rep. 2022 May 11;12:7777. doi: 10.1038/s41598-022-11883-5 (PMC9095662; doi:10.1038/s41598-022-11883-5)
Supplement: Supplementary file 1 — Supplementary Figure 1. [file 41598_2022_11883_MOESM1_ESM.docx]

**Supplementary Fig. 1.** Deformation evolution of monolayer MoS_2_ membrane under uniaxial tension at 1K in the armchair direction for various porosities. (a) porosity of 1.56%, (b) porosity of 4.69%, (c) porosity of 10.94%.
