# Supplementary material for: Thermal and mechanical characterization of nanoporous two-dimensional MoS2 membranes
Source: Sci Rep. 2022 May 11;12:7777. doi: 10.1038/s41598-022-11883-5 (PMC9095662; doi:10.1038/s41598-022-11883-5)
Supplement: Supplementary file 4 — Supplementary Figure 4. [file 41598_2022_11883_MOESM4_ESM.docx]

**Supplementary Fig. 4.** The failure of monolayer MoS_2_ membrane under the uniaxial tension along the zigzag direction at different temperatures.
