# Supplementary material for: Thermal and mechanical characterization of nanoporous two-dimensional MoS2 membranes
Source: Sci Rep. 2022 May 11;12:7777. doi: 10.1038/s41598-022-11883-5 (PMC9095662; doi:10.1038/s41598-022-11883-5)
Supplement: Supplementary file 7 — Supplementary Figure 7. [file 41598_2022_11883_MOESM7_ESM.docx]

**Supplementary Fig. 7.** (**a-b**) The relation between the thermal conductivity and the length of the monolayer MoS_2_ membrane in the armchair and zigzag directions with various porosities. (**c-d**) The relation between *1/κ* and *1/L* of monolayer MoS_2_ membrane in the armchair and zigzag directions with various porosities.
