# Supplementary material for: Thermal and mechanical characterization of nanoporous two-dimensional MoS2 membranes
Source: Sci Rep. 2022 May 11;12:7777. doi: 10.1038/s41598-022-11883-5 (PMC9095662; doi:10.1038/s41598-022-11883-5)
Supplement: Supplementary file 8 — Supplementary Table 1. [file 41598_2022_11883_MOESM8_ESM.docx]

**Supplementary Table 1.** Comparison of the calculated results with the relevant literature.

| Material | Young’s modulus  (GPa) | Ultimate stress (GPa) | Failure strain (%) | Thermal conductivity (W/m-K) | Temperature | Method | References |
| --- | --- | --- | --- | --- | --- | --- | --- |
| MoS_2_ | 159.05 (armchair)  161.95 (zigzag) | 15.03  14.95 | 12.3  12.9 | - | 300 K |  | Changli et al. [79] |
| MoS_2_ | 158.1 (armchair)  159.7 (zigzag) | 16.5  16.2 | 19.6  22.7 | - | 1 K |  | Subad et al. [29] |
| MoS_2_ | 165.7 (armchair)  167.0 (zigzag) | 16.8  16.2 | 19.5  20 | - | 1 K |  | Jiang et al. [46] |
| MoS_2_ | 148.9÷248.3 |  | 23 | - | 0 K |  | Cooper et al. [80] |
| MoS_2_ |  |  |  | 47.5 | 300 K | DFT | Xiang et al. [35] |
| MoS_2_ |  |  |  | 23.2 | 300 K | DFT | Cai et al. [38] |
| MoS_2_ |  |  |  | 130.3 | 300 K | DFT | Ouyang et al. [81] |
| MoS_2_ |  | - | - | 51.4 | 300 K | MD | Krishnamoorthy et al. [51] |
| MoS_2_ |  |  |  | 42.18 | 300 K | MD | Chen et al. [55] |
| MoS_2_ |  |  |  | 80-105 | 300 K | Experiment | Yang et al. [30] |
| MoS_2_ |  |  |  | 40.8 | 300 K | Experiment | Li et al. [31] |
| MoS_2_ |  |  |  | 34.5±4 | 300 K | Experiment | Yan et al. [32] |
| MoS_2_ |  |  |  | 84±17 | 300 K | Experiment | Zhang et al. [34] |
| MoS_2_ |  | - | - | 52 | 300 K | Experiment | Shahoo et al. [52] |
| MoS_2_ |  |  |  | 48-52 | 300 K | Experiment | Jo et al. [54] |
| MoS_2_ | 158.73 (armchair)  158.01 (zigzag) | 16.96  16.25 | 19.78  22.79 |  | 1 K | MD | This research |
|  | 149.08 (armchair) | 13.54 | 12.4 | *κ_MD_ =* 52.91  *κ_corr_ =* 43.41 | 300 K | MD | This research |
|  | 145.04 (zigzag) | 12.90 | 13.23 | *κ_MD_ =*52.35  *κ_corr_ =* 42.92 | 300 K |  |  |

**Supplementary References:**

[79] Yi, C., Hu, C., Bai, M., Lv, J., & Tang, D. (2020). Molecular dynamics study on the mechanical properties of multilayer MoS_2_ under different potentials. *Nanotechnology*, *31*(21), 215703.

[80] Cooper, R. C., Lee, C., Marianetti, C. A., Wei, X., Hone, J., & Kysar, J. W. (2013). Nonlinear elastic behavior of two-dimensional molybdenum disulfide. *Physical Review B*, *87*(3), 035423.

[81] Ouyang, B., Chen, S., Jing, Y., Wei, T., Xiong, S., & Donadio, D. (2018). Enhanced thermoelectric performance of two dimensional MS_2_ (M= Mo, W) through phase engineering. *Journal of Materiomics*, *4*(4), 329-337.
